# Characteristics, mechanism, and management of pain in atopic dermatitis: A literature review

**DOI:** 10.1002/clt2.12079

**Published:** 2021-12-02

**Authors:** Jia‐Xin Li, Rui‐Jia Dong, Yue‐Ping Zeng

**Affiliations:** ^1^ Department of Dermatology State Key Laboratory of Complex Severe and Rare Diseases Peking Union Medical College Hospital Chinese Academy of Medical Science and Peking Union Medical College, National Clinical Research Center for Dermatologic and Immunologic Diseases Beijing China; ^2^ Peking Union Medical College Chinese Academy of Medical Sciences Beijing China; ^3^ Department of Plastic Surgery, Beijing Tsinghua Changgung Hospital, School of Clinical Medicine Tsinghua University Beijing China

**Keywords:** atopic dermatitis, epidemiology, management, mechanism, pain, atopische dermatitis, epidemiologie, management, mechanismus, schmerzen

## Abstract

**Background:**

Atopic dermatitis (AD) is a chronic, pruritic, immune‐mediated inflammatory disease. Developments in basic science and clinical research have increased our understanding of AD. Although pain as a symptom of AD is underemphasized in previous studies, multiple researchers address pain as a frequent burden of AD. However, the exact role of pain in AD is not fully understood.

**Aims:**

Our review aimed to summarize the current evidence focusing on characteristics, mechanism, and management of pain in AD.

**Materials & Methods:**

We conducted a thorough literature review in the PubMed database to figure out different aspects discussing pain in AD, including pain symptoms, burden, the relationship between pain and itch, mechanism, and pain management in AD.

**Results and Conclusion:**

AD patients affected by skin pain vary from 42.7%‐92.2% with remarkable intensity and heavy burden. Skin pain and itch interacted both in symptoms and mechanisms. Atopic skin with the impaired barrier, neurogenic inflammation mediators, peripheral and central sensitization of pain may possibly explain pain mechanism in AD. Future research is needed to clarify the commonality and disparity of pain and itch in AD in order to seek efficacious medications and treatment.

## 1 INTRODUCTION

Atopic dermatitis (AD) is one of the most common chronic skin diseases and is characterized by itch and inflammatory eczematous lesions. This condition not only affects children (20%) but also is prevalent in adults (10%), especially in developed countries.^1^ Impairment in physical or psychosocial activities is caused by AD, resulting in, for example, sleep disturbances, depression, or anxiety, all of which place an enormous economic burden on health resources.^2^ Epidermal barrier disruptions and skin inflammation mutually reinforce AD pathophysiological processes. A consequence of the interaction of a compromised barrier with the type 2 immune environment is the promotion of percutaneous allergic sensitization.^3^ In addition, AD may increase the risk of asthma, allergic rhinitis, or other immune‐mediated inflammatory diseases. Although itch is the most prominent symptom, pain has recently been considered a nonnegligible burden for AD.^4^

As defined by Samuel Hafenreffer, itch is an unpleasant sensation associated with the desire or reflex to scratch. The definition of pain promulgated by the international association is that pain is a distressing experience associated with actual or potential tissue damage with sensory, emotional, cognitive, and social components.^5^

A thorough search of the literature was performed in the PubMed database using (atopic dermatitis OR eczema) AND (pain) as search terms. There was an obvious increase from 147 articles before 2000 to 808 articles until 2021, reflecting aroused interest in clinical practice. After adding “clinical study” as the article type, 86 articles were found, and finally, we included 3 original articles related to pain in AD. The rules for excluding irrelevant publications were AD without pain description or pain symptoms with other types of dermatitis. Additional articles were retrieved through the hand‐searching of reference lists.

## 2 EPIDEMIOLOGY OF AD

The prevalence of AD is increasing,^6^ and the prevalence and severity of the disease vary in different regions and ethnic populations.^7^, ^8^ In particular, 10.7%–20% children currently suffer from AD.^8^, ^9^, ^10^ The prevalence of adult AD was reported to be 4.9% in the United States, 3.5% in Canada, 4.4% in the EU, 2.1%–6.9% in Japan, and 2.6% in Korea, ranging from 2.6%–6.9% worldwide.^11^, ^12^, ^13^

Although AD is usually characterized as a childhood disease, persistence of AD from children to adults is common, particularly in those with early onset in infancy.^14^ With a 34.1% lifetime prevalence of AD, nearly half (10%–17.1%) of AD adults continuously suffered from a schooltime age diagnosis of AD, as reported in a Denmark prospective study.^15^ Although child‐onset AD is more prevalent, adult‐onset AD is not uncommon, noted as 11%–13% in some countries.^16^

## 3 PAIN IS A COMMON SYMPTOM IN AD

Although itch is the predominant and most common feature of AD, the symptom of pain is emphasized as more frequently encountered than perceived. However, pain symptoms, unlike itch, have not been assessed thoroughly. Sometimes, itch and pain appear at the same time, making it hard to distinguish their respective impacts. However, half of the AD patients (50.4%) in one study were able to distinguish between the two symptoms.^17^ A total of 59%–78% of AD patients have been reported to present with undistinguished and accompanied pain and itch.^18^, ^19^

**FIGURE 1:**
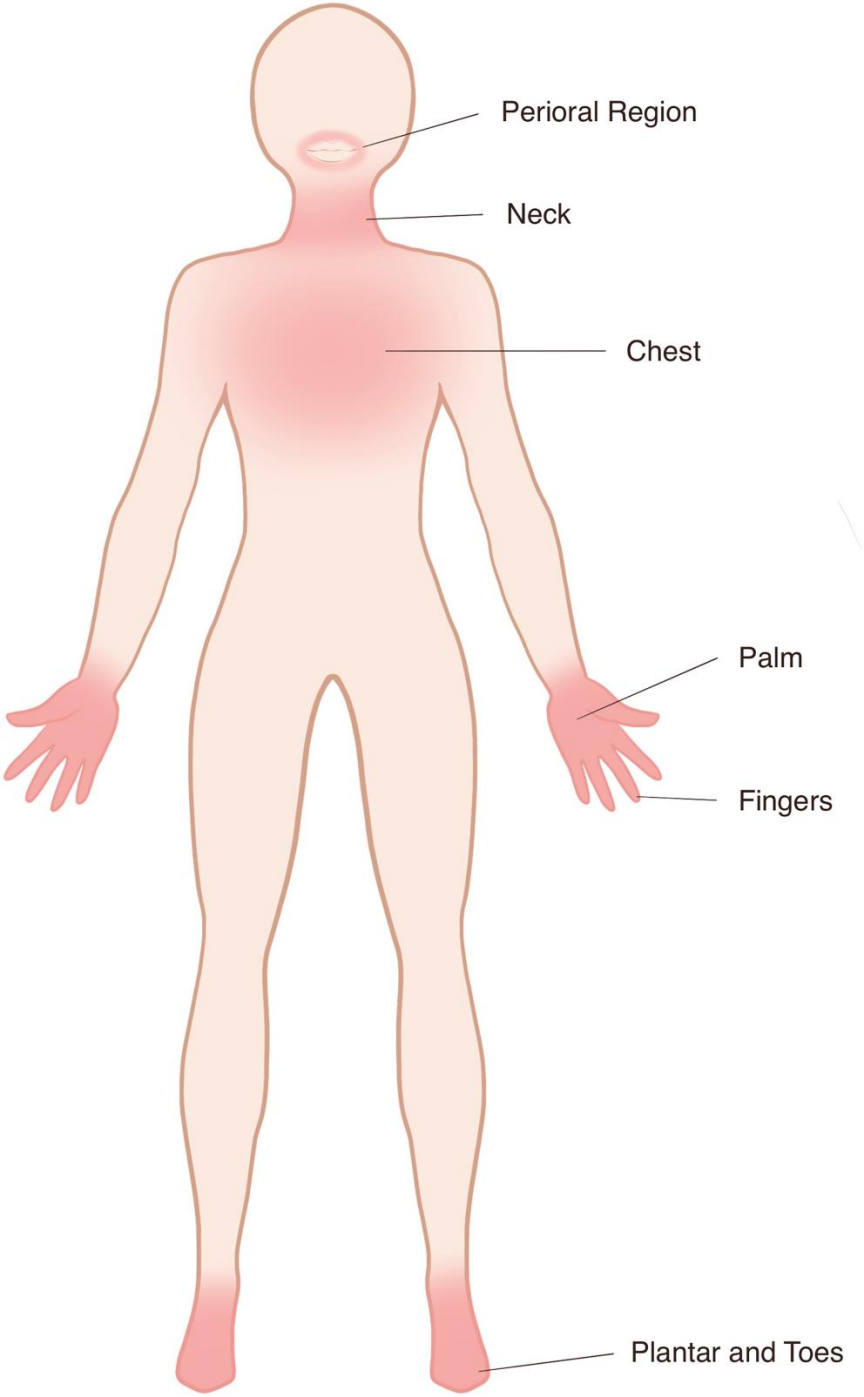
Areas with greater pain burden. The greater pain burdens usually occur on the hands, perioral regions, plantar and toes, neck, and chest where cutaneous sensory nerve distribution is denser

Various evaluations of itch in AD patients are applied in practice; however, pain is often inadequately estimated.^20^ Although few studies have paid attention to pain symptom in the past, to update the findings, skin pain is commonly experienced by a great proportion of AD patients, ranging from 42.7% to 92.2%.^17^, ^21^, ^22^, ^23^, ^24^ The McGill questionnaire is used to evaluate pain from five different dimensions.^24^ Greater pain burden usually occurred on the hands, perioral regions, plantar and toes, neck, and chest, where the cutaneous sensory nerve distribution is denser (Figure 1).^18^, ^25^

Considering the description of skin pain among AD patients, burning (71.4%) was the most commonly used descriptor, followed by prickling (65.2%). Additionally, tingling (25.6%), electric shock sensation (25.6%), and cold pain sensation (14.3%) were less likely to be experienced.^22^ A recent survey by Pojawa‐Gołąb and Reich provided similar results, showing that most AD patients (77.2%) complained about burning/stinging pain, and a great amount of them (60.7%) considered this pain annoying. Some AD patients (29.4%) described it as a sharp pain.^17^

To characterize the frequency, intensity, characteristics, and associations of pain in AD patients, Silverberg et al.^26^ performed a cross‐sectional, Internet survey‐based study of 602 adults in the US population. The results showed that 61% (365/602) reported pain from AD, 33% (199/602) experienced pain at least once per week, and 5% (30/602) reported pain daily. Pain was mostly associated with scratching (27%) and fissures in the skin (27%), followed by inflamed red skin (25%) or minor burning from creams or ointments (10%). More specific pain characteristics of AD are summarized in Table 1.

**TABLE 1:** The review of pain characteristics in AD from relevant references

References	Patient characteristics	AD patients affected by skin pain (%)	Skin pain severity	Driver of skin pain	Characteristics of pain	Associated topical morphologic characteristics	Other burdens
Vakharia et al.^23^	305 patients (age: 13–97 years)	42.7	Severe or very severe pain (13.8%)	Itch (16.8%); scratch (11.2%); both (72%)	Throbbing, biting, stinging, burning, sharp, tingling, pinprick‐like, and crawling	Excoriations	Poor QOL, sleep disturbance, and mental health symptoms
Maarouf et al.^18^	103 patients (age: 5–74 years)	78	–	Itch (61%); scratch with wound (51%); scratch without wound (60%)	Burning (73%), stinging (57%), soreness (42%), tightness (42%), tenderness (39%), and throbbing (32%)	Red (88%); cracked (73%); dry (72%); scaling (57%); crusting/scabbing (56%); swollen (54%); bleeding (51%); warm (51%); weeping/oozing (42%);	Sleep interference (80%) and leisure activity impairment (78%)
Silverberg et al.^26^	602 patients (age: 52.0 ± 16.3 years)	61	Moderate or severe pain (3.7%)	Scratch (27%), fissure (27%), inflamed red skin (25%), and burning from ointments (10%)	–	–	Increased self‐consciousness; negative impacts on shopping, playing sports, working, or studying; and treatment burden

Abbreviation: AD, atopic dermatitis.

The level of pain is closely correlated with the severity of AD, such that pain frequency, pain intensity, and pain score represent prompts for disease evaluation.^25^, ^26^ Misery et al.^22^ used DN4 (douleur neuropathique—four questions) to illustrate that 57.5% of AD patients suffered from neuropathic pain (DN4i > 3). The intensity of pain was estimated by VAS_mean_ 5.3 ± 2.9.^21^ Among a French sample of 5000 AD patients, more than half declared skin pain, and the intensity was reported to be approximately 6–10.^27^ Recently, a novel Numeric Rating Scale (NRS) evaluation for pain among 463 adult AD patients based on questionnaires and skin examination was confirmed to be valid for clinical practice.^28^ Although ceiling effects existed, this study would help reflect the severity of the disease. Additionally, with the distinct longitudinal course of AD skin pain, AD patients suffer long‐term skin pain even when under treatment. Furthermore, the baseline level of pain appeared to predict the toughness of skin pain.^29^

## 4 BURDENS OF SKIN PAIN

Skin pain is significantly associated with impaired quality of life (QOL) using tools, such as the Dermatology Life Quality Index. Patients who had both severe itch and severe pain were likely to obtain higher scores on these measurements than patients with either of these features.^30^, ^31^ A recent study also noted skin pain in 3834 AD patients, which interestingly reported correlated increased levels of joint pain.^32^

Subjective symptoms accompanying AD can negatively influence patients’ well‐being, such as impaired social life, economic burden, and sleep disturbance. The coexistence of insomnia was 82% in AD patients. Sleep disturbance also crucially affects both adults and children, especially those with severe AD in adults together with early onset and persistent AD in children.^33^, ^34^ Newer cross‐sectional data involving 2893 adults with AD showed that 79.7% of the group had difficulties sleeping during the past 3 days, and sleep disturbance strongly impacted QOL, mental health, and other outcomes.^35^ Although itch contributes a more substantial association with insomnia and sleep quality, pain is not a trivial contributor to these burdens.^21^

Skin pain may increase mental health burdens. Clinical observations have shown that patients with chronic pain exhibit symptoms of increased anxiety, depression, and cognitive impairment.^36^ The reason is that pain is a stressor that increases autonomic sympathetic activity and perceived stress.^37^ Conversely, anxiety and stress have been implicated in pain hyperalgesia,^38^ where the coexistence of pain and mood disorders may form a vicious cycle leading to a worsening disease prognosis and QOL.

Clinicians must further understand the unique burden of pain in AD patients to best evaluate clinical severity and QOL interference. Skin pain should also be regularly assessed and could be monitored as a treatment response.

## 5 SYMPTOMATIC RELATIONSHIP BETWEEN PAIN AND ITCH

Studies have suggested that pain is related to scratching, fissures, and/or inflamed red skin, which is mostly because of itch.^39^ Scratching instantly relieves the itch sensation through the activation of pain‐sensory fibers that can inhibit itch sensation. This explains why people are prone to scratch if they feel itchy, and an itch–scratch cycle develops.^40^ Furthermore, stimulation of thin cutaneous nerve fibers can induce an analgesic effect,^41^ implying that scratching may also reduce pain at the same time. Scratching is like adding insult to injury, exacerbating the already dysfunctional skin barrier. Subsequently, more pain and itch sensations are produced. What is still unknown is whether the pain originates from AD (the disease itself), or simply ensues from itch, or both.^42^

Vakharia et al.^23^ reported that 16.8% of painful AD patients thought that the skin pain was part of their itch, while 11.2% thought the pain was a result of scratching, and 72.0% thought both. Patients with skin pain were more likely to describe their itch using terms that resembled neuropathic pain. Moreover, skin pain was positively associated with AD severity and itch; the close observed relationship between itch and pain highlights the potential benefits of ameliorating skin pain in AD treatment.^25^

## 6 MECHANISM OF PAIN IN AD

Itch and pain are closely related, sharing similar pathological conditions but manifesting distinct sensations. However, pain processing in the skin is still not clearly understood in terms of the mechanism. Schmelz^43^ summarized that three abnormal processes would result in pain and itch processing in AD, namely, decreased protective factors resulting in destruction of the skin barrier, increased levels of excitatory skin mediators and peripheral neuron sensitization, the last two of which cause activation of peripheral neurons in AD.

### 6.1 Atopic skin and scratching

Sensitive skin is defined as “unpleasant sensations occurrence in response to stimuli that normally should not provoke such sensations.”^44^ Dry skin and atopic predisposition may correlate with sensitive skin.^45^ Research has suggested an association between sensitive skin and an impaired skin barrier, with up to 80% of AD patients declaring sensitive skin.^46^, ^47^ This finding indicates weakened integrity and stability of the epidermal skin barrier in AD patients, followed by failure of nerve ending protection, and environmental agent penetration develops sensitive skin.^46^ Therefore, AD individuals are more likely to feel pain and burning sensations even though the stimuli are not strong under common conditions. Furthermore, scratching enhances itch sensations, producing an “itch‐scratch‐itch” cycle.^48^ It is difficult to distinguish chronic pain and itch, suggesting common interacting mechanisms in between.

### 6.2 Neurogenic inflammation

Peripheral mediators, especially inflammatory mediators, contribute to pain. Lysophosphatidic acid, cathepsin S (CatS), and IL‐33, noted as inflammatory mediators, have implicit relationships with chronic pain conditions.^43^ CatS activates CD4+ T cells, resulting in neuropathic pain.^49^ In particular, IL‐33 has been demonstrated to boost inflammation and pain in some diseases.^50^ These inflammatory mediators are reported to be overexpressed in AD.^51^, ^52^ Therefore, these results suggest that a high level of inflammatory mediators in AD activates peripheral neuron pathways.

In addition, as summarized by Liu and Ji,^53^ compared with itch processing, cytokines including IL‐1β and IL‐6 together with chemokines, such as chemokine C–C motif ligand 2 (CCL2), CCL5, and C–X–C motif chemokine (CXCL1) could induce pain.

Nerve growth factor (NGF) serves as a key substance in chronic pain conditions by primary pain afferents.^48^ The level of NGF was confirmed to be increased in AD patients.^54^ Evidence has also revealed the clinical relief of pain symptom by anti‐NGF studies.^55^

### 6.3 Peripheral sensitization to pain

Inflammatory mediators activate nociceptive neurons in the pain signaling pathway. Considering peripheral sensitization to pain, G protein‐coupled receptors (GPCRs) and transient receptor potential (TRP) channels, both remarkable receptor families, contribute to skin sensation.^56^ Via peripheral sensitization, these receptors become activated and can transmit pain stimuli signals for further pathways.

TRP vanilloid 1 (TRPV1), TRP ankyrin 1 (TRPA1), and transient receptor melastatin member 8 (TRPM8) are TRP channels that are detected at high levels in AD lesions.^57^, ^58^ Activation of TRPV1 results in increased levels of proinflammatory neuropeptide substance P, accelerating neurogenic inflammation.^57^ Mas‐related GPCRs (Mrgprs), serving as members of the GPCR superfamily, are expressed by primary sensory neurons for peripheral skin sensations.^59^, ^60^ Mas‐related GPCR D (MrgprD) is likely to be involved in neuropathic pain mechanisms as a nociceptor and mediate pain signaling.^61^, ^62^ Moreover, β‐alanine‐responsive QC fibers activate MrgprD expression, resulting in capsaicin pain.^63^

Nociceptors become stimulated by mass fibers and signal the process of pain and itch. Pain neurons and touch neurons can sense skin stimuli and conduct pain until the brain. However, these receptors can also inhibit the signaling pathways of itch by GABAergic interneurons and glycine interneurons.^43^

### 6.4 Central sensitization to pain

The endothelin‐1/endothelin receptor type B (ET‐1/EDNRB) pathway was implicated in an atopic mouse model to explain the correlation between neuropathic pain and allergic inflammation, as also supported by an enhanced level of ET‐1 in atopic patients.^64^ This study implied that ET‐1/EDNRB mediates spinal astroglia and microglial activation in AD, inducing neuropathic pain.^64^ Conclusively, peripheral sensation and activation of the nervous system would transmit the pain signal. The activation of spinal microglia boosts the expression of cytokines including IL‐1β and TNF‐α and brain‐derived neurotrophic factor.^53^, ^65^ Moreover, the activation of astroglia results in the increased release of CCL2.^66^ Consequently, these mediators regulate pain processing and central sensitization.

In addition to the evidence above, chronic pain‐associated brain areas are activated in the anterior cingulate cortex, insula, and dorsolateral prefrontal cortex of the brain in AD patients, as observed by brain imaging.^67^

### 6.5 Pain and itch

Itch and pain share largely overlapping mediators and receptors such as TRPV1, TRPA1, TLRs, and PARs, both in physiological and pathological conditions. Itch stimuli can be perceived as pain in some chronic neuropathic pain conditions.^68^ While differences between acute pain and acute itch are striking, chronic pain and chronic itch share many similar mechanisms, including peripheral sensitization, central sensitization, loss of inhibitory control in the spinal cord, and neuroimmune interactions at peripheral and central levels.^69^

On the one hand, pain inhibits itch. Some studies of hydrogen peroxide showed that pruritogens at higher doses in rodents produce pain to suppress itch.^53^ Another study showed that glutamate released from the central branches of nociceptors not only triggers pain transmission but also activates inhibitory neurons for itch suppression.^70^ Research on the skin of the normal population has shown that itch can be significantly reduced by applying conditioning pain.^71^ These findings parallel the condition that after scratching (regarded as a kind of pain stimulus), itch is reduced to some extent. For the opposing role of pain suppressing itch, inhibition of pain may produce itch in turn.^72^ This possibility is partly supported by the process by which pain‐inhibiting opioids generate itch.^73^

On the other hand, however, under pathological conditions, such as AD, painful stimuli may not be sufficient to suppress itch, whereas they may instead enhance itch in these chronic itch patients.^42^ Some painful stimuli may evoke itch in patients with dermatitis but not in normal people. Taking bradykinin as an example, which evoked only weak pain in nonlesional or normal skin, it was observed to induce intense itch in lesional skin, indicating that simultaneous pain stimuli increase itch conversely.^74^ Existing studies found that cutaneous innervation is reduced in pruritic skin, as itch receptors are unregulated to drive ongoing itch.^75^ This hypersensitization may lead us to wonder whether pain sensation is upregulated simultaneously.

As comprehensively theorized based on clinical observations, pain and itch processes are differentiated by spatial and temporal patterns, originally caused by neuropathy and inflammation, as mentioned before.^43^ It remains controversial whether pain‐killing treatment would aggravate itch, although it is widely accepted that some drugs for treating chronic pain are also effective in chronic itch because of the interlinking mechanism. Rather than separating itch and pain, research concepts should address the overlapping mechanisms as chronic inflammatory conditions.

## 7 MANAGEMENT OF PAIN IN AD

The overwhelming majority of AD therapies have been proven effective in treating annoying itch; however, pain is always poorly studied and understood. Skin pain may be useful as a marker of treatment progress and disease severity since it introduces indispensable burdens. Nonetheless, the relationships of skin pain with disease severity, anatomical location, and the use of pain medication are still unclear and require further investigation.

To examine analgesic medication in AD patients, Thyssen et al.^25^ performed a cross‐sectional study of 3208 randomly selected adults from the general population and 3834 adults with a diagnosis of AD in Denmark. Skin pain was positively associated with AD severity and itch, while pain medication (including paracetamol, nonsteroidal anti‐inflammatory drugs, and opioids) was not significantly increased. Other drugs, such as gabapentin^76^ and pregabalin^77^ have been used in patients with severe AD.^25^ In addition to paracetamol, tramadol, or other analgesic, there are some other treatments for alleviating pain, for example, treatments, such as vagus nerve stimulation^78^ and acupuncture,^79^ but clinical application of these treatments in AD patients has not yet been reported. Nevertheless, analgesic and gabapentinoid treatment requires a comprehensive evaluation of patient age, medical history, pain intensity, and drug effects.^80^

The principal therapy to manage pain and itch is topical therapy.^80^ The use of emollients is considered to prevent lesions from becoming painful.^81^ There is still a lack of evidence to confirm pain relief by many AD treatments. Moreover, some topical treatments for AD may even evoke pain sensation. When the lesion is worsened by infections, which would promote pain processing, specialized antimicrobials and antiseptics are recommended for treatment.^82^

Topical therapies for AD include topical corticosteroids (TCSs), calcineurin inhibitors (TCIs), and new topical treatments, such as topical phosphodiesterase 4 (PDE4) inhibitors, novel topical Janus kinase (JAK) inhibitors, tofacitinib and delgocitinib, and UV therapy. Systemic treatments have provided limited evidence regarding pain relief of patients.^80^ Most of the treatments present great effects in reducing itch; however, few papers have reported diminishment of pain in AD patients. It is still unknown whether TCSs reduce pain.

Calcineurin inhibitors are related to a reduction in pain hypersensitivity.^80^ For example, FK506 (tacrolimus) is an immunosuppressant widely used as an ointment in the treatment of AD. Local application of FK506 might evoke burning sensations, and some patients have felt pain after using FK506 topically. The underlying mechanism might be explained by FK506 mediating TRPA1 activation.^83^ To lower the side effects of FK506, a microemulsion system integrating FK506 into a menthol/camphor eutectic might be helpful, owing to their cooling nature in topical analgesics.^84^

However, recent studies have provided new perspectives on biological treatments related to pain induction. JAK inhibitors have been shown to palliate pain. Baricitinib, a JAK inhibitor, was reported to reduce patient‐reported pain in AD patients, with a remarkable decrease in NRS responders, and to further enhance their QOL.^85^ Additionally, significant amelioration of pain symptom in moderate‐to‐severe AD was observed after dupilumab (IL‐4Rα mAb) treatment.^86^

One problem is that doctors fail to ask AD patients about skin pain symptom, which has aroused public attention, as 79.4% of AD patients did not receive help from physicians due to a lack of solicitude.^17^ As advocated by Fuxench,^4^ the assessment of pain represents a research hotspot needing better understanding.

Therefore, to address the complaint of pain among AD patients, the primary treatment is to apply AD therapy supplemented with emollients, general principles and education, and psychological assistance. If pain persistently exists, several therapies can aid the situation, including analgesics, gabapentinoids, antidepressants, anti‐JAK, anti‐PDE4, and μ‐opioids.^80^

In summary, the higher prevalence of pain in AD than previously thought and the close but not fully determined relationship between itch and pain highlight the potential benefit of establishing more sound treatments to ameliorate itch and relieve skin pain.

## 8 CONCLUSIONS

Skin pain in AD has been characterized as an increasingly remarkable symptom. Clinicians and health policy experts should be aware of the related characteristics between pain and itch. Future research is needed to clarify the commonality and disparity of these two apparent symptoms in order to seek efficacious medications.

## CONFLICT OF INTEREST

We declare that we have no conflicts of interest.

## References

[1] Legat FJ . Itch in atopic dermatitis – what is new? Front Med. 2021;8:644760. 10.3389/fmed.2021.644760 PMC8137993 34026782

[2] Nutten S . Atopic dermatitis: global epidemiology and risk factors. Ann Nutr Metab. 2015;66 (suppl 1):8‐16. 25925336 10.1159/000370220

[3] Weidinger S , Novak N . Atopic dermatitis. Lancet. 2016;387(10023):1109‐1122. 26377142 10.1016/S0140-6736(15)00149-X

[4] Fuxench ZCC . Pain in atopic dermatitis: it's time we addressed this symptom further. Br J Dermatol. 2020;182(6):1326–1327. 31956992 10.1111/bjd.18785

[5] International Association for the Study of Pain . The need of a taxonomy. Pain. 1979;6(3):247‐252.460931

[6] Mayba JN , Gooderham MJ . Review of atopic dermatitis and topical therapies. J Cutan Med Surg. 2017;21(3):227‐236. 28300440 10.1177/1203475416685077

[7] Mei‐Yen Yong A , Tay YK . Atopic dermatitis: racial and ethnic differences. Dermatol Clin. 2017;35(3):395‐402. 28577807 10.1016/j.det.2017.02.012

[8] Mallol J , Crane J , von Mutius E , et al. The International Study of Asthma and Allergies in Childhood (ISAAC) phase three: a global synthesis. Allergol Immunopathol. 2013;41(2):73‐85. 10.1016/j.aller.2012.03.001 22771150

[9] Asher MI , Montefort S , Björkstén B , et al. Worldwide time trends in the prevalence of symptoms of asthma, allergic rhinoconjunctivitis, and eczema in childhood: ISAAC phases one and three repeat multicountry cross‐sectional surveys. Lancet. 2006;368(9537):733‐743. 16935684 10.1016/S0140-6736(06)69283-0

[10] Shaw TE , Currie GP , Koudelka CW , Simpson EL . Eczema prevalence in the United States: data from the 2003 National Survey of Children’s Health. J Invest Dermatol. 2011;131(1):67‐73. 20739951 10.1038/jid.2010.251 PMC3130508

[11] Barbarot S , Auziere S , Gadkari A , et al. Epidemiology of atopic dermatitis in adults: results from an international survey. Allergy. 2018;73(6):1284‐1293. 29319189 10.1111/all.13401

[12] Saeki H , Tsunemi Y , Fujita H , et al. Prevalence of atopic dermatitis determined by clinical examination in Japanese adults. J Dermatol. 2006;33(11):817‐819. 17074002 10.1111/j.1346-8138.2006.00187.x

[13] Kim MJ , Kang TW , Cho EA , et al. Prevalence of atopic dermatitis among Korean adults visiting health service center of the Catholic Medical Center in Seoul Metropolitan Area, Korea. J Korean Med Sci. 2010;25(12):1828‐1830. 21165305 10.3346/jkms.2010.25.12.1828 PMC2995244

[14] Abuabara K , Yu AM , Okhovat JP , Allen IE , Langan SM . The prevalence of atopic dermatitis beyond childhood: a systematic review and meta‐analysis of longitudinal studies. Allergy. 2018;73(3):696‐704. 28960336 10.1111/all.13320 PMC5830308

[15] Mortz CG , Andersen KE , Dellgren C , Barington T , Bindslev‐Jensen C . Atopic dermatitis from adolescence to adulthood in the TOACS cohort: prevalence, persistence and comorbidities. Allergy. 2015;70(7):836‐845. 25832131 10.1111/all.12619

[16] Giampiero Patriarca M , Claudio D’Ambrosio M , Domenico Schiavino M , Luigi Maria Larocca M , Eleonora Nucera M , Alessandro Milani M . Clinical usefulness of patch and challenge tests in the diagnosis of cell‐mediated allergy to betalactams. Ann Allergy Asthma Immunol. 1999;83(3):257‐266. 10507273 10.1016/S1081-1206(10)62650-6

[17] Pojawa‐Gołąb M , Reich A . Skin pain in patients with atopic dermatitis or psoriasis: a web‐based survey. Acta Derm Venereol. 2020;100(16):adv00258. 32830283 10.2340/00015555-3617 PMC9234986

[18] Maarouf M , Kromenacker B , Capozza KL , et al. Pain and itch are dual burdens in atopic dermatitis. Dermatitis. 2018;29(5):278‐281. 30179978 10.1097/DER.0000000000000406

[19] Dawn A , Papoiu AD , Chan YH , Rapp SR , Rassette N , Yosipovitch G . Itch characteristics in atopic dermatitis: results of a web‐based questionnaire. Br J Dermatol. 2009;160(3):642‐644. 19067703 10.1111/j.1365-2133.2008.08941.x

[20] Terwee CB , Bot SD , de Boer MR , et al. Quality criteria were proposed for measurement properties of health status questionnaires. J Clin Epidemiol. 2007;60(1):34‐42. 17161752 10.1016/j.jclinepi.2006.03.012

[21] Kaaz K , Szepietowski JC , Matusiak L . Influence of itch and pain on sleep quality in atopic dermatitis and psoriasis. Acta Derm Venereol. 2019;99(2):175‐180. 30307027 10.2340/00015555-3065

[22] Misery L , Saint Aroman M , Zkik A , et al. Chronic pain in patients with skin disorders. Acta Derm Venereol. 2017;97(8):986‐988. 28498388 10.2340/00015555-2694

[23] Vakharia PP , Chopra R , Sacotte R , et al. Burden of skin pain in atopic dermatitis. Ann Allergy Asthma Immunol. 2017;119(6):548‐552. 29223299 10.1016/j.anai.2017.09.076 PMC5726579

[24] Yosipovitch G , Goon AT , Wee J , Chan YH , Zucker I , Goh CL . Itch characteristics in Chinese patients with atopic dermatitis using a new questionnaire for the assessment of pruritus. Int J Dermatol. 2002;41(4):212‐216. 12031029 10.1046/j.1365-4362.2002.01460.x

[25] Thyssen JP , Halling‐Sonderby AS , Wu JJ , Egeberg A . Pain severity and use of analgesic medication in adults with atopic dermatitis: a cross‐sectional study. Br J Dermatol. 2020;182(6):1430‐1436. 31556099 10.1111/bjd.18557

[26] Silverberg JI , Gelfand JM , Margolis DJ , et al. Pain is a common and burdensome symptom of atopic dermatitis in United States adults. J Allergy Clin Immunol Pract. 2019;7(8):2699‐2706. 31228619 10.1016/j.jaip.2019.05.055

[27] Huet F , Shourick J , Séité S , Taïeb C , Misery L . Pain in atopic dermatitis: an online population‐based survey. Acta Derm Venereol. 2020;100(14):adv00198. 32424431 10.2340/00015555-3521 PMC9199926

[28] Silverberg JI . Validity and reliability of a novel numeric rating scale to measure skin‐pain in adults with atopic dermatitis. Arch Dermatol Res. 2021;313(10):855‐861. 33547939 10.1007/s00403-021-02185-3

[29] Hong MR , Lei D , Yousaf M , Chavda R , Gabriel S , Silverberg JI . A real‐world study of the longitudinal course of skin pain in adult atopic dermatitis. J Am Acad Dermatol. 2021. 10.1016/j.jaad.2021.04.021 33872718

[30] Bridgman AC , Block JK , Drucker AM . The multidimensional burden of atopic dermatitis: an update. Ann Allergy Asthma Immunol. 2018;120(6):603‐606. 29555350 10.1016/j.anai.2018.03.009

[31] Grant L , Seiding Larsen L , Trennery C , et al. Conceptual model to illustrate the symptom experience and humanistic burden associated with atopic dermatitis in adults and adolescents. Dermatitis. 2019;30(4):247‐254. 31261226 10.1097/DER.0000000000000486 PMC6641086

[32] Egeberg A , Griffiths CEM , Williams HC , Andersen YMF , Thyssen JP . Clinical characteristics, symptoms and burden of psoriasis and atopic dermatitis in adults. Br J Dermatol. 2020;183(1):128‐138. 31630393 10.1111/bjd.18622

[33] McKenzie C , Paller AS , Fishbein A , Silverberg JI . Association between the longitudinal course of AD, sleep disturbance, and overall health in US children. J Allergy Clin Immunol Pract. 2020;8(2):812‐814. 31472295 10.1016/j.jaip.2019.08.027 PMC7391260

[34] Li JC , Fishbein A , Singam V , et al. Sleep disturbance and sleep‐related impairment in adults with atopic dermatitis: a cross‐sectional study. Dermatitis. 2018;29(5):270‐277. 30234614 10.1097/DER.0000000000000401 PMC6169311

[35] Silverberg JI , Chiesa‐Fuxench Z , Margolis D , et al. Sleep disturbances in atopic dermatitis in US adults. Dermatitis. 2021. 10.1097/DER.0000000000000731 33675326

[36] Belinskaia DA , Belinskaia MA , Barygin OI , Vanchakova NP , Shestakova NN . Psychotropic drugs for the management of chronic pain and itch. Pharmaceuticals. 2019;12(2):99. 10.3390/ph12020099 PMC6631469 31238561

[37] Bjorkedal E , Flaten MA . Expectations of increased and decreased pain explain the effect of conditioned pain modulation in females. J Pain Res. 2012;5:289‐300. 23049277 10.2147/JPR.S33559 PMC3442740

[38] Benedetti F , Amanzio M , Vighetti S , Asteggiano G . The biochemical and neuroendocrine bases of the hyperalgesic nocebo effect. J Neurosci. 2006;26(46):12014‐12022. 17108175 10.1523/JNEUROSCI.2947-06.2006 PMC6674855

[39] Kusari A , Han AM , Schairer D , Eichenfield LF . Atopic dermatitis: new developments. Dermatol Clin. 2019;37(1):11‐20. 30466683 10.1016/j.det.2018.07.003

[40] Mack MR , Kim BS . The itch‐scratch cycle: a neuroimmune perspective. Trends Immunol 2018;39(12):980‐991. 30471983 10.1016/j.it.2018.10.001 PMC8896504

[41] Nilsson HJ , Schouenborg J . Differential inhibitory effect on human nociceptive skin senses induced by local stimulation of thin cutaneous fibers. Pain. 1999;80(1‐2):103‐112. 10204722 10.1016/s0304-3959(98)00205-x

[42] Andersen HH , Yosipovitch G , Arendt‐Nielsen L . Pain inhibits itch, but not in atopic dermatitis? Ann Allergy Asthma Immunol. 2018;120(5):548‐549. 29729727 10.1016/j.anai.2017.12.025

[43] Schmelz M . Itch processing in the skin. Front Med. 2019;6:167. 10.3389/fmed.2019.00167 PMC6659104 31380380

[44] Misery L , Ständer S , Szepietowski JC , et al. Definition of sensitive skin: an expert position paper from the special interest group on sensitive skin of the International Forum for the Study of Itch. Acta Derm Venereol. 2017;97(1):4‐6. 26939643 10.2340/00015555-2397

[45] Misery L , Jourdan E , Huet F , et al. Sensitive skin in France: a study on prevalence, relationship with age and skin type and impact on quality of life. J Eur Acad Dermatol Venereol. 2018;32(5):791‐795. 29397030 10.1111/jdv.14837

[46] Misery L , Loser K , Ständer S . Sensitive skin. J Eur Acad Dermatol Venereol. 2016;30((Suppl 1)):2‐8. 26805416 10.1111/jdv.13532

[47] Yatagai T , Shimauchi T , Yamaguchi H , et al. Sensitive skin is highly frequent in extrinsic atopic dermatitis and correlates with disease severity markers but not necessarily with skin barrier impairment. J Dermatol Sci. 2018;89(1):33‐39. 29122406 10.1016/j.jdermsci.2017.10.011

[48] Li C , Kim HJ , Back SK , Na HS . Common and discrete mechanisms underlying chronic pain and itch: peripheral and central sensitization. Pflugers Arch. 2021;473(10):1603‐1615. 34245379 10.1007/s00424-021-02599-y

[49] Zhang X , Wu Z , Hayashi Y , Okada R , Nakanishi H . Peripheral role of cathepsin S in Th1 cell‐dependent transition of nerve injury‐induced acute pain to a chronic pain state. J Neurosci. 2014;34(8):3013‐3022. 24553941 10.1523/JNEUROSCI.3681-13.2014 PMC6608526

[50] Fattori V , Borghi SM , Verri WA, Jr . IL‐33/ST2 signaling boosts inflammation and pain. Proc Natl Acad Sci USA. 2017;114(47):E10034‐E10035. 29109297 10.1073/pnas.1716120114 PMC5703330

[51] Imai Y . Interleukin‐33 in atopic dermatitis. J Dermatol Sci. 2019;96(1):2‐7. 31455506 10.1016/j.jdermsci.2019.08.006

[52] Kim N , Bae KB , Kim MO , et al. Overexpression of cathepsin S induces chronic atopic dermatitis in mice. J Invest Dermatol. 2012;132(4):1169‐1176. 22170489 10.1038/jid.2011.404

[53] Liu T , Ji RR . Oxidative stress induces itch via activation of transient receptor potential subtype ankyrin 1 in mice. Neurosci Bull. 2012;28(2):145‐154. 22466125 10.1007/s12264-012-1207-9 PMC3339413

[54] Yamaguchi J , Aihara M , Kobayashi Y , Kambara T , Ikezawa Z . Quantitative analysis of nerve growth factor (NGF) in the atopic dermatitis and psoriasis horny layer and effect of treatment on NGF in atopic dermatitis. J Dermatol Sci. 2009;53(1):48‐54. 18922683 10.1016/j.jdermsci.2008.08.011

[55] Yeh JF , Akinci A , Al Shaker M , et al. Monoclonal antibodies for chronic pain: a practical review of mechanisms and clinical applications. Mol Pain. 2017;13:1744806917740233. 29056066 10.1177/1744806917740233 PMC5680940

[56] Mollanazar NK , Smith PK , Yosipovitch G . Mediators of chronic pruritus in atopic dermatitis: getting the itch out? Clin Rev Allergy Immunol. 2016;51(3):263‐292. 25931325 10.1007/s12016-015-8488-5

[57] Hutter MM , Wick EC , Day AL , et al. Transient receptor potential vanilloid (TRPV‐1) promotes neurogenic inflammation in the pancreas via activation of the neurokinin‐1 receptor (NK‐1R). Pancreas. 2005;30(3):260‐265. 15782105 10.1097/01.mpa.0000153616.63384.24

[58] Oh MH , Oh SY , Lu J , et al. TRPA1‐dependent pruritus in IL‐13‐induced chronic atopic dermatitis. J Immunol. 2013;191(11):5371‐5382. 24140646 10.4049/jimmunol.1300300 PMC4175413

[59] Dong X , Han S , Zylka MJ , Simon MI , Anderson DJ . A diverse family of GPCRs expressed in specific subsets of nociceptive sensory neurons. Cell. 2001;106(5):619‐632. 11551509 10.1016/s0092-8674(01)00483-4

[60] McNeil B , Dong X . Peripheral mechanisms of itch. Neurosci Bull. 2012;28(2):100‐110. 22466121 10.1007/s12264-012-1202-1 PMC5560392

[61] Zylka MJ , Rice FL , Anderson DJ . Topographically distinct epidermal nociceptive circuits revealed by axonal tracers targeted to Mrgprd. Neuron. 2005;45(1):17‐25. 15629699 10.1016/j.neuron.2004.12.015

[62] Wang C , Gu L , Ruan Y , et al. Facilitation of MrgprD by TRP‐A1 promotes neuropathic pain. FASEB J. 2019;33(1):1360‐1373. 30148678 10.1096/fj.201800615RR PMC6988841

[63] Wooten M , Weng HJ , Hartke TV , et al. Three functionally distinct classes of C‐fibre nociceptors in primates. Nat Commun. 2014;5:4122. 24947823 10.1038/ncomms5122 PMC4072246

[64] Yamasaki R , Fujii T , Wang B , et al. Allergic inflammation leads to neuropathic pain via glial cell activation. J Neurosci. 2016;36(47):11929‐11945. 27881779 10.1523/JNEUROSCI.1981-16.2016 PMC6604914

[65] Ji RR , Suter MR . p38 MAPK, microglial signaling, and neuropathic pain. Mol Pain. 2007;3:33. 17974036 10.1186/1744-8069-3-33 PMC2186318

[66] Gao YJ , Zhang L , Samad OA , et al. JNK‐induced MCP‐1 production in spinal cord astrocytes contributes to central sensitization and neuropathic pain. J Neurosci. 2009;29(13):4096‐4108. 19339605 10.1523/JNEUROSCI.3623-08.2009 PMC2682921

[67] Ishiuji Y , Coghill RC , Patel TS , Oshiro Y , Kraft RA , Yosipovitch G . Distinct patterns of brain activity evoked by histamine‐induced itch reveal an association with itch intensity and disease severity in atopic dermatitis. Br J Dermatol. 2009;161(5):1072‐1080. 19663870 10.1111/j.1365-2133.2009.09308.x PMC2784001

[68] Baron R , Schwarz K , Kleinert A , Schattschneider, Jr. , Wasner G . Histamine‐induced itch converts into pain in neuropathic hyperalgesia. Neuroreport. 2001;12(16):3475‐3478. 11733694 10.1097/00001756-200111160-00020

[69] Granot M , Yakov S , Ramon M . Enhanced itch intensity is associated with less efficient descending inhibition processing for itch but not pain attenuation in chronic dermatology patients. Pain Med 2020;21(10):2538‐2545. 31642915 10.1093/pm/pnz263

[70] Liu Y , Abdel Samad O , Zhang L , et al. VGLUT2‐dependent glutamate release from nociceptors is required to sense pain and suppress itch. Neuron. 2010;68(3):543‐556. 21040853 10.1016/j.neuron.2010.09.008 PMC2991105

[71] Andersen HH , van Laarhoven AIM , Elberling J , Arendt‐Nielsen L . Modulation of itch by conditioning itch and pain stimulation in healthy humans. J Pain. 2017;18(12):1437‐1450. 28709954 10.1016/j.jpain.2017.07.002

[72] Ikoma A , Steinhoff M , Stander S , Yosipovitch G , Schmelz M . The neurobiology of itch. Nat Rev Neurosci. 2006;7(7):535‐547. 16791143 10.1038/nrn1950

[73] Schmelz M . Itch and pain differences and commonalities. Handb Exp Pharmacol. 2015;227:285‐301. 25846624 10.1007/978-3-662-46450-2_14

[74] Hosogi M , Schmelz M , Miyachi Y , Ikoma A . Bradykinin is a potent pruritogen in atopic dermatitis: a switch from pain to itch. Pain. 2006;126(1‐3):16‐23. 16842920 10.1016/j.pain.2006.06.003

[75] Tan Y , Ng WJ , Lee SZX , et al. 3‐dimensional optical clearing and imaging of pruritic atopic dermatitis and psoriasis skin reveals downregulation of epidermal innervation. J Invest Dermatol. 2019;139(5):1201‐1204. 30471253 10.1016/j.jid.2018.11.006

[76] Solak Y , Biyik Z , Atalay H , et al. Pregabalin versus gabapentin in the treatment of neuropathic pruritus in maintenance haemodialysis patients: a prospective, crossover study. Nephrology. 2012;17(8):710‐717. 22909343 10.1111/j.1440-1797.2012.01655.x

[77] Ahuja RB , Gupta GK . A four arm, double blind, randomized and placebo controlled study of pregabalin in the management of post‐burn pruritus. Burns. 2013;39(1):24‐29. 23089176 10.1016/j.burns.2012.09.016

[78] Kirchner A , Stefan H , Schmelz M , Haslbeck KM , Birklein F . Influence of vagus nerve stimulation on histamine‐induced itching. Neurology. 2002;59(1):108‐112. 12105316 10.1212/wnl.59.1.108

[79] Napadow V , Li A , Loggia ML , et al. The brain circuitry mediating antipruritic effects of acupuncture. Cereb Cortex. 2014;24(4):873‐882. 23222890 10.1093/cercor/bhs363 PMC3948489

[80] Misery L , Belloni Fortina A , El Hachem M , et al. A position paper on the management of itch and pain in atopic dermatitis from the International Society of Atopic Dermatitis (ISAD)/Oriented Patient‐Education Network in Dermatology (OPENED) Task Force. J Eur Acad Dermatol Venereol. 2021;35(4):787‐796. 33090558 10.1111/jdv.16916

[81] Cristaudo A , Lupi F , Mariano M , Cianchini G , De Rocco M , De Pità O . Clinical and instrumental evaluation of the efficacy of an emollient cream and a cleansing cream in the management of mild to moderate adulthood atopic dermatitis. G Ital Dermatol Venereol. 2018;153(6):855‐859. 30518177 10.23736/S0392-0488.18.06078-9

[82] Wollenberg A , Barbarot S , Bieber T , et al. Consensus‐based European guidelines for treatment of atopic eczema (atopic dermatitis) in adults and children: part II. J Eur Acad Dermatol Venereol. 2018;32(6):850‐878. 29878606 10.1111/jdv.14888

[83] Kita T , Uchida K , Kato K , Suzuki Y , Tominaga M , Yamazaki J . FK506 (tacrolimus) causes pain sensation through the activation of transient receptor potential ankyrin 1 (TRPA1) channels. J Physiol Sci. 2019;69(2):305‐316. 30478741 10.1007/s12576-018-0647-z PMC10717736

[84] Wang Y , Cao S , Yu K , et al. Integrating tacrolimus into eutectic oil‐based microemulsion for atopic dermatitis: simultaneously enhancing percutaneous delivery and treatment efficacy with relieving side effects. Int J Nanomedicine. 2019;14:5849‐5863. 31440050 10.2147/IJN.S212260 PMC6679700

[85] Thyssen JP , Buhl T , Fernández‐Peñas P , et al. Baricitinib rapidly improves skin pain resulting in improved quality of life for patients with atopic dermatitis: analyses from BREEZE‐AD1, 2, and 7. Dermatol Ther. 2021;11(5):1599‐1611. 10.1007/s13555-021-00577-x PMC8484387 34275122

[86] Silverberg JI , Simpson EL , Guttman‐Yassky E , et al. Dupilumab Significantly modulates pain and discomfort in patients with atopic dermatitis: a post hoc analysis of 5 randomized clinical trials. Dermatitis. 2021;32(1S):S81‐S91. 33165005 10.1097/DER.0000000000000698 PMC8560147

